# Silage additives improve fermentation quality, aerobic stability and rumen degradation in mixed silage composed of amaranth and corn straw

**DOI:** 10.3389/fpls.2023.1189747

**Published:** 2023-06-22

**Authors:** Jian Ma, Xue Fan, Zhuang Ma, Xiuwen Huang, Minghuan Tang, Fuquan Yin, Zhihui Zhao, Shangquan Gan

**Affiliations:** ^1^ College of Coastal Agricultural Sciences, Guangdong Ocean University, Zhanjiang, China; ^2^ College of Animal Science, Xinjiang Agricultural University, Urumchi, China

**Keywords:** amaranth, corn straw, silage additive, fermentation quality, rumen degradability

## Abstract

The objective of this research was to investigate effects of different additives on the fermentation quality, aerobic stability and rumen degradation of mixed silage composed of amaranth and corn straw. The mixture ratio of amaranth to corn straw was 78%: 22%. Three additives were selected in this study and five groups were as follows: control group (CON, without additive), lactic acid bacteria group (LAB, 5 mg/kg, *Lactobacillus plantarum* ≥ 1.6×10^10^ CFU/g and *L. buchneri* ≥ 4.0×10^9^ CFU/g), glucose group (GLU, 30 g/kg), cellulase group (CEL, 2 mg/kg) and lactic acid bacteria, glucose and cellulase group (LGC, added at the same levels as in individual group). The period of ensiling was 60 days. Fermentation quality, chemical composition and aerobic stability of mixed silage were analyzed. Four cows with permanent ruminal fistula were selected as experimental animals. Nylon bag technique was used to study rumen degradation characteristic of dry matter (DM), crude protein (CP), neutral detergent fiber (NDF) and acid detergent fiber (ADF) of mixed silage. Compared with CON group, the addition of different silage additives could improve mixed silage quality of amaranth and corn straw to some extent. Combining three additives significantly increased (*P* < 0.05) the DM, CP and lactic acid contents, whereas decreased (*P* < 0.05) the ADF and NDF contents as well as pH and ammonia nitrogen/total nitrogen. Moreover, the aerobic stability and rumen degradation of DM, CP and NDF were significantly improved (*P* < 0.05) in LGC group when compared to other groups. In conclusion, the combined addition of lactic acid bacteria, glucose and cellulase increased DM, CP and lactic acid contents as well as lactic acid bacteria count, decreased NDF and ADF contents and aerobic bacteria and mold counts, improved aerobic stability and rumen degradation of amaranth and corn straw mixed silage.

## 1 Introduction

In dairy cows’ production, the roughages (e.g. alfalfa hay, Chinese wildrye and corn silage) can provide essential nutrients for animals, which usually account for 30~70% in the ration (NRC, 2001). In order to preserve the quality of roughage, silage is commonly used in dairy farming. Corn is one of primary crops for ensiling; however, compared with other silage such as alfalfa, the contents of crude protein (CP) (<10% dry matter [DM] basis) and rumen degradable nutrients in corn silage are lower (Fernandes et al., 2022). In addition, due to the limited land resources, water scarcity and poor soils, the yield of some crops (e.g. corn and sorghum) is limited in some parts of the world (Jacobsen, 2014). Thus, it is of great importance in dairy farming by making full use of various roughage resources. In recent years, the utilization of non-conventional feed resource with high CP content, digestibility and yield has attracted increasing attention.

Some crops that can adapt to water shortage, high temperature and poor soils can be used as feedstuff resources for ruminants’ industry under certain harsh conditions (Li et al., 2018b; Taghipour et al., 2021). Amaranth (*Amaranthus hypochondriacus*) is one of such crops. As a C_4_ dicotyledonous crop, amaranth can grow in the areas with poor soils, water shortage and high temperature (Sarmadi et al., 2016). According to the survey, the yield of amaranth can reach up to 85 t/ha (fresh weight) and 16 t/ha (DM) (Abbasi et al., 2012). Moreover, compared with corn, the amaranth has a higher CP concentration (approximately 25%, DM basis), a lower lignin content (approximately 4%, DM basis) (Aderibigbe et al., 2022) and lower concentrations of oxalic acid and nitrate (Rahjerdi et al., 2015). In ruminants’ production, the partial substitution of amaranth silage for maize silage in the ration of dairy cows (Rezaei et al., 2015) and fattening lambs (Rezaei et al., 2014) does not affect animal health and performance.

Our previous research found that the optimal growth stage of amaranth was from peak flowering stage to heading stage for ensiling (Ma et al., 2019). We also found that the fresh amaranth moisture content is high and water soluble carbohydrate (WSC) is low. The contents of moisture and WSC are key factors to determine the silage quality (Zi et al., 2022b). In China, the corn straw is rich in resources and the DM content of corn straw is high. In this study, we used amaranth and corn straw as raw materials for ensiling. On the other hand, adding silage additives can improve the fermentation characteristics and nutritional value of silage, especially for grass silage (Li et al., 2022). The lactic acid bacteria can promote the fermentation process by consuming WSC to produce lactic acid and inhibit the growth of harmful bacteria, then improve the alfalfa silage quality (Li et al., 2018a). In hybrid *Pennisetum* silage, the cellulase inoculation can improve fermentation quality by degrading structural carbohydrates to provide fermentation substrate (Xiong et al., 2022). In the current study, we selected lactic acid bacteria, glucose and cellulase as silage additives. For ruminants, an important parameter of nutritional value evaluation in roughage is the ruminal degradation rate of nutrients (Stirling et al., 2022). Generally, the nylon bag technology is utilized to evaluate the ruminal degradation rate of feedstuffs (Diao et al., 2020). Therefore, this study was conducted to evaluate the effects of different additives on the fermentation quality, chemical composition, aerobic stability and ruminal degradation characteristics of mixed silage composed of amaranth and corn straw.

## 2 Materials and methods

### 2.1 Experimental field and preparation of silage

The amaranth was planted in the experimental field (covers an area of 800 m^2^, 41°25′ E longitude and 88°40′ N latitude) at the Heshuo County, Bayingolin Mongol Autonomous Prefecture. The altitude of this area is approximately 2217 m, with annual average temperature and rainfall of 11.4°C and 58.6 mm respectively. The soil at the experimental field is sandy clay and the pH is approximately 7.8. During the growth stage of amaranth in 2021 (May to October), the rain capacity was 30.3 mm. Before sowing, the nitrogenous (400 kg/ha) and potassic (60 kg/ha) fertilizers were provided in the amaranth field as base fertilizers. The nitrogen fertilizer was urea (N content was 46%) and the potassium fertilizer was potassium chloride (K content was 62%). The seeding rate was approximately 0.8 kg/ha.

The amaranth seeds were sown manually on the 10th of May (2021) and harvested at heading stage. The whole-plants was cut to a 5-cm stubble height with reaping hook. Then, the harvested fresh amaranth and corn straw were chopped into fragments of 1.5 to 2 cm in length using a forage chopper (Zhoushi Shengzhuoxin Machinery Processing Factory, Suzhou, Jiangsu, China) before making silage. In the current study, according to the principle that the water content of mixed silage materials was about 65%, the mixture ratio of amaranth and corn straw was 78%: 22%. The chemical compositions of amaranth and corn straw are presented in **Supplementary Table S1**.

### 2.2 Experimental design

In this study, lactic acid bacteria (*Lactobacillus plantarum* ≥ 1.6×10^10^ CFU/g and *L. buchneri* ≥ 4.0×10^9^ CFU/g, Silage Legend Technology Co., LTD., Hohhot, Inner Mongolia, China), glucose (99% purity, Shengxing Chemical Co., LTD., Jining, Shandong, China) and cellulase (5000 U/g, Xiasheng Biotechnology Co., LTD., Beijing, China) were used as silage additives. The five treatments were as follows: (1) control group without any additive (CON); (2) ensiled amaranth and corn straw inoculated with lactic acid bacteria (LAB, added level was 5 mg/kg); (3) mixed silage supplementation with glucose (GLU, added level was 30 g/kg); (4) mixed silage supplementation with cellulase (CEL, added level was 2 mg/kg) (5) lactic acid bacteria, glucose and cellulase group (LGC, the dose was same as that added separately). All the additives were mixed into water and then evenly sprayed onto the silage materials. The CON group was sprayed with equivalent water. Subsequently, the mixed amaranth and corn straw were tightly compacted and sealed in a fermentation container (2 L capacity) to make silage. Each treatment had four replicates. The fermentation containers were stored at the laboratory. After 60 days of fermentation, the containers were opened, and samples were collected for analysis of chemical composition, fermentation quality, aerobic stability and ruminal degradability.

### 2.3 Chemical component, fermentation quality, microbial composition and aerobic stability analysis

The matured silage samples were weighed and dried at 65°C in a forced-air oven for 48 h to a constant weight. Next, the air-dried samples were ground to pass through a 1-mm sieve (Taifeng Machinery Equipment Co., LTD., Yantai,Shandong, China). Subsequently, the samples were used to determine the contents of DM (105 °C), CP (No. 988.05) and OM (No. 942.02) reference to the AOAC procedure (AOAC, 2006). The NDF and ADF concentrations of silage samples were analyzed according to the methods described by Van Soest et al. (1991).

Fresh mixed silage of 20 g from each container was blended with 180 mL distilled water and stored at 4 °C. Samples were leached for 24 h and filtered through four layers of gauze. Then, the pH value was determined by pH meter (Ruizhen Electronic Technology Co., Ltd., Shanghai, China). The WSC concentration was measured using anthrone colorimetry (Hansen and Møller, 1975). Total nitrogen (TN) was analyzed via a nitrogen analyzer (Youpu General Technology Co., LTD., Beijing, China) and phenol- hypochlorite method was used to measure ammonia nitrogen (NH_3_-N) (Broderick and Kang, 1980). In addition, the contents of organic acids, including lactic acid, acetic acid, propionic acid and butyric acid, were analyzed using high-performance liquid chromatography (APS8026, DE Aupos Scientific) as described by Chen et al. (2022).

Plate count method was used to count the lactic acid bacteria, yeast, aerobic bacteria, mold and coliform bacteria in the mixed silage. The 10 g fresh silage samples of each container were mixed with 90 mL sterilized water, and serially diluted to enumerate the microbial composition in a sterile solution. The number of lactic acid bacteria, yeast, aerobic bacteria, mold and coliform bacteria was determined according to the procedure described by Sun et al. (2021).

After 60 days of fermentation, the mixed silage was conducted to 5 days aerobic stability experiment reference to procedure described by He et al. (2020). In the current experiment, the numbers of lactic acid bacteria, yeast, aerobic bacteria, mold and coliform bacteria were used as spoilage parameters. Furthermore, the aerobic stability time was defined that the silage temperature exceeded the environmental temperature above 2 °C. The temperature was collected using a multipoint real-time temperature recorder (Mike Sensor Technology Co., LTD., Hangzhou, Zhejiang, China).

### 2.4 Ruminal nutrient degradability

In this study, four Holstein cows (560.2 ± 13.8 kg of body weight; dry period) with a permanent ruminal fistula were selected to determine the ruminal degradability of DM, CP, NDF and ADF using the nylon bag technology. All animals were fed the total mixed ration which was formulated according to the NRC (NRC, 2001). The dietary ratio of concentrate and roughage was 40:60. The feed ingredients and nutrient contents of diet are shown in **Supplementary Table S2**. Cows were regularly provided diet twice daily at 08:00 and 17:00 and had free access to water during the experiment. All cows had 20 days to adapt the diet.

The nylon bag was sewed to 8 × 12 cm with a pore size of 50 μm and the air-dried silage samples were smashed through a 4-mm sieve. Five grams of samples were accurately weighed and sealed into nylon bags. Then, the nylon bags were fixed in the soft rubber hose and placed into the nylon net. Nylon net was put into the rumen through the permanent ruminal fistula before morning feeding and the other end of net was fixed to the fistula. In the rumen of cows, the silage samples were incubated for 4, 8, 16, 24, 36, 48 and 72 h. At each time point, the samples had 4 replicates.

After serially taking out the bags at the corresponding time point, the nylon bag was rinsed using cold tap-water until the outlet water was clear. Next, the bags were dried in a forced-air oven (65°C) to a constant weight. Residues were weighted and smashed via a micromill to pass a 1-mm screen sieve, and used to determine the nutrient contents (DM, CP, NDF and ADF) according to the methods mentioned earlier. The ruminal degradability (P) of nutrients at each time (t) was estimated using an exponential curve as P = a + b (1 − e^−ct^) and the effective degradability (ED) of nutrients was calculated by ED (%) = a + (b × c)/(c + k) according to previous reference (Øskov and McDonald, 1979). In the above-mentioned equations, “a” is the rapidly degradable fraction of samples to be tested; “b” is the insoluble but potentially degradable fraction that degrades at a constant fractional rate (c); “e” is the base of natural logarithm; “k” is the rumen outflow rate. A fixed outflow rate of “k” was 0.031/h according to our previous study (Ma et al., 2019). The values of a, b and c were calculated by the non-linear regression program of SAS software (version 9.2).

### 2.5 Statistical analysis

Data were analyzed by one-way ANOVA procedure of the SPSS statistical software (version 20.0). Duncan test was used to determine the differences among groups. Data were presented as means and standard error of mean (SEM). The significance level was indicated at *P* < 0.05, and 0.05 ≤ *P* < 0.10 represented a tendency.

## 3 Results

### 3.1 Chemical composition of mixed silage

The DM content of LAB and LGC groups was higher (*P* < 0.05) than that of CON, GLU and CEL groups (**Table 1**). LGC group showed highest CP content, which was increased by 20.34% as compared to CON group (*P* < 0.05). No significant difference (*P* > 0.05) of OM concentration was found among all groups. Compared with CON and GLU groups, the NDF and ADF contents of CEL and LGC groups were significantly decreased (*P* < 0.05). In addition, the WSC concentration of GLU group was higher (*P* < 0.05) than CON and LAB groups.

**Table 1:** Effects of different treatments on the chemical composition of mixed silage (DM basis, %).

Items	Treatments					SEM	*P*-value
	CON	LAB	GLU	CEL	LGC		
DM	24.93^b^	29.56^a^	25.12^b^	25.42^b^	30.69^a^	0.679	0.001
CP	5.85^b^	6.76^a^	6.44^ab^	6.27^ab^	7.04^a^	0.139	0.048
OM	86.19	87.06	85.84	85.46	87.43	0.582	0.842
NDF	60.04^a^	56.20^ab^	58.87^a^	54.10^b^	53.13^b^	0.829	0.015
ADF	39.96^a^	38.43^ab^	41.08^a^	34.75^b^	35.43^b^	0.737	0.006
WSC	2.19^b^	1.82^b^	2.90^a^	2.26^ab^	2.24^ab^	0.117	0.038

CON, control group without additive; LAB, mixed silage inoculated with lactic acid bacteria 5 mg/kg; GLU, mixed silage supplementation with glucose 30 g/kg; CEL, mixed silage supplementation with cellulase 2 mg/kg; LGC, mixed silage supplementation with lactic acid bacteria 5 mg/kg, glucose 30 g/kg and cellulase 2 mg/kg.

DM, dry matter; CP, crude protein; OM, organic matter; NDF, neutral detergent fiber; ADF, acid detergent fiber; WSC, water soluble carbohydrate.

In the same row, values with different letter mean significant differences (*P* < 0.05).

### 3.2 Fermentation quality of mixed silage

As shown in **Table 2**, the pH of CEL and LGC groups was lower (*P* < 0.05) than that of CON group. The NH_3_-N/TN of LGC group was minimum and lower (*P* < 0.05) than CON, LAB and GLU groups. On the contrary, LGC group displayed highest lactic acid content, which was increased by 58.36%, 21.26%, 13.06% and 18.40% respectively as compared with CON, LAB, GLU and CEL groups (*P* < 0.05). The contents of acetic and propionic acids in CON group were higher (*P* < 0.05) than those in GLU, CEL and LGC groups. No butyric acid was detected in LAB, GLU and LGC groups. Additionally, compared with CON, LAB and CEL groups, the lactic acid/acetic acid of LGC group was significantly increased (*P* < 0.05).

**Table 2:** Effects of different treatments on the fermentation characteristics of mixed silage.

Items	Treatments					SEM	*P*-value
	CON	LAB	GLU	CEL	LGC		
pH	4.16^a^	4.04^ab^	3.94^abc^	3.89^bc^	3.78^c^	0.042	0.026
NH_3_-N/TN	4.76^a^	3.80^b^	3.60^b^	3.04^c^	2.87^c^	0.159	<0.001
Lactic acid (%, DM)	3.17^c^	4.14^b^	4.44^b^	4.24^b^	5.02^a^	0.153	<0.001
Acetic acid (%, DM)	2.03^a^	1.73^ab^	1.33^bc^	1.36^bc^	1.11^c^	0.094	0.003
Propionic acid (%, DM)	0.032^a^	0.011^bc^	0.019^b^	0.016^bc^	0.007^c^	0.002	0.001
Butyric acid (%, DM)	0.003	ND	ND	0.002	ND	0.001	0.141
Lactic acid/Acetic acid	1.59^c^	2.40^bc^	3.52^ab^	3.31^b^	4.77^a^	0.306	0.002

CON, control group without additive; LAB, mixed silage inoculated with lactic acid bacteria 5 mg/kg; GLU, mixed silage supplementation with glucose 30 g/kg; CEL, mixed silage supplementation with cellulase 2 mg/kg; LGC, mixed silage supplementation with lactic acid bacteria 5 mg/kg, glucose 30 g/kg and cellulase 2 mg/kg.

DM, dry matter; NH_3_-N, ammonia nitrogen; TN, total nitrogen; ND, not detected.

In the same row, values with different letter mean significant differences (*P* < 0.05).

### 3.3 Microbial population of mixed silage

**Figure 1:**
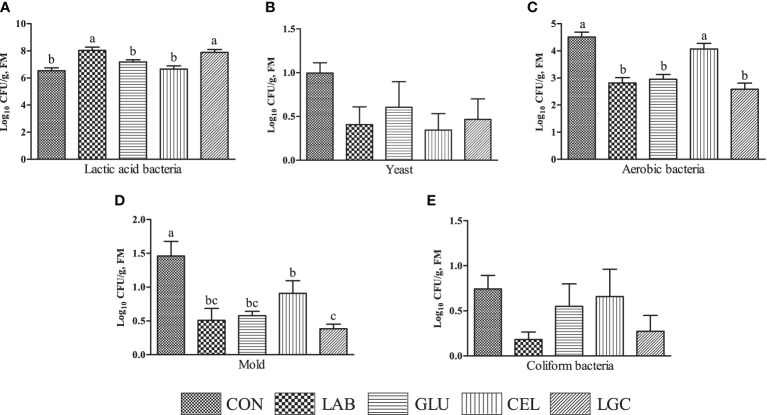
Effects of different treatments on the microbial population of mixed silage. **(A)** lactic acid bacteria; **(B)** yeast; **(C)** aerobic bacteria; **(D)** mold; **(E)** coliform bacteria. CON, control group without additive; LAB, mixed silage inoculated with lactic acid bacteria 5 mg/kg; GLU, mixed silage supplementation with glucose 30 g/kg; CEL, mixed silage supplementation with cellulase 2 mg/kg; LGC, mixed silage supplementation with lactic acid bacteria 5 mg/kg, glucose 30 g/kg and cellulase 2 mg/kg. FM, fresh matter. Columns with different superscript letters mean significant differences (*P* < 0.05).

Notably, the number of lactic acid bacteria in LAB and LGC groups was higher (*P* < 0.05) than other groups (**Figure 1A**)). There was no significant difference (*P* > 0.05) of yeast (**Figure 1B**) and coliform bacteria (**Figure 1E**) counts among all treatments. However, compared with LAB, GLU and LGC groups, the aerobic bacteria count in CON and CEL groups was significantly increased (*P* < 0.05) (**Figure 1C**). All the inoculated treatments exhibited significantly decreased (*P* < 0.05) mold count (**Figure 1D**).

### 3.4 Aerobic stability of mixed silage

**Figure 2:**
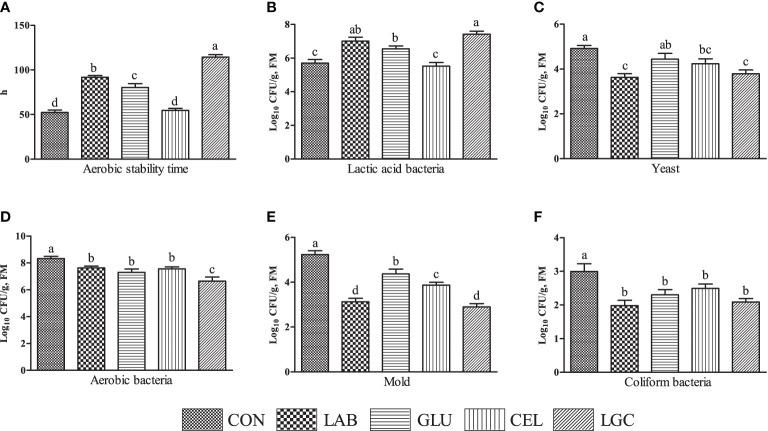
Effects of different treatments on the aerobic stability of mixed silage. **(A)** aerobic stability time; **(B)** lactic acid bacteria; **(C)** yeast; **(D)** aerobic bacteria; **(E)** mold; **(F)** coliform bacteria. CON, control group without additive; LAB, mixed silage inoculated with lactic acid bacteria 5 mg/kg; GLU, mixed silage supplementation with glucose 30 g/kg; CEL, mixed silage supplementation with cellulase 2 mg/kg; LGC, mixed silage supplementation with lactic acid bacteria 5 mg/kg, glucose 30 g/kg and cellulase 2 mg/kg. FM, fresh matter. Columns with different superscript letters mean significant differences (*P* < 0.05).

After aerobic exposure for 5 days, except for CEL group, other inoculated treatments significantly extended (*P* < 0.05) the aerobic stability time of mixed silage compared with CON group (**Figure 2A**). The LAB, GLU and LGC groups showed significantly higher (*P* < 0.05) lactic acid bacteria count than CON and CEL groups (**Figure 2B**). Compared with CON group, the yeast count was reduced by 26.22% and 22.97% in LAB and LGC groups respectively (*P* < 0.05) (**Figure 2C**). The numbers of aerobic bacteria, mold and coliform bacteria in inoculated treatments were lower (*P* < 0.05) than those in CON group (**Figures 2D-F**). In addition, the aerobic bacteria and mold counts of LGC group was significantly decreased (*P* < 0.05) as compared with GLU and CEL groups (**Figures 2D, E**).

### 3.5 Ruminal dry matter degradation of mixed silage

The ruminal DM degradation of LGC group at 72 h was higher (*P* < 0.05) than that of CON and GLU groups (**Table 3**). At 4 h and 8 h, the LGC and LAB groups exhibited higher (*P* < 0.05) DM degradation as compared with CON group. The DM degradation velocity of all mixed silage was faster before 24 h and then tended to be stable. The rapidly degradable fraction of LAB and LGC groups was higher (*P* < 0.05) than other groups (**Table 4**). Besides, the LGC group had maximum slowly degradable fraction and higher than (*P* < 0.05) CON, GLU and CEL groups. Compared with CON and GLU groups, the DM effective degradability of LGC group was significantly increased (*P* < 0.05).

**Table 3:** Rumen degradability of dry matter of mixed silage at different time points (%).

Time point (h)	Treatments					SEM	*P*-value
	CON	LAB	GLU	CEL	LGC		
4	26.61^b^	31.98^a^	28.60^ab^	29.57^ab^	33.33^a^	0.818	0.049
8	32.65^b^	38.69^a^	36.25^ab^	35.91^ab^	41.69^a^	1.002	0.035
16	42.31	47.57	47.99	44.98	51.05	1.049	0.072
24	53.10	55.89	56.14	53.11	59.34	1.052	0.320
36	58.35	61.16	59.28	59.93	63.91	0.811	0.232
48	62.51	65.40	64.52	65.76	68.11	0.798	0.279
72	64.34^c^	70.14^ab^	66.56^bc^	67.33^abc^	71.94^a^	0.874	0.027

CON, control group without additive; LAB, mixed silage inoculated with lactic acid bacteria 5 mg/kg; GLU, mixed silage supplementation with glucose 30 g/kg; CEL, mixed silage supplementation with cellulase 2 mg/kg; LGC, mixed silage supplementation with lactic acid bacteria 5 mg/kg, glucose 30 g/kg and cellulase 2 mg/kg.

In the same row, values with different letter mean significant differences (*P* < 0.05).

**Table 4:** Rumen degradation parameters of dry matter of mixed silage.

Items	Treatments					SEM	*P*-value
	CON	LAB	GLU	CEL	LGC		
a (%)	22.56^b^	26.55^a^	23.18^b^	24.20^b^	26.67^a^	0.460	<0.001
b (%)	39.75^c^	45.39^ab^	42.62^bc^	41.74^c^	46.64^a^	0.701	0.002
c (%/h)	0.035^d^	0.037^cd^	0.046^b^	0.053^a^	0.040^c^	0.002	<0.001
a + b (%)	62.31^b^	71.94^a^	65.80^b^	65.94^b^	73.31^a^	1.059	<0.001
ED (%)	43.47^c^	51.26^ab^	48.49^b^	50.56^ab^	52.87^a^	0.828	<0.001

CON, control group without additive; LAB, mixed silage inoculated with lactic acid bacteria 5 mg/kg; GLU, mixed silage supplementation with glucose 30 g/kg; CEL, mixed silage supplementation with cellulase 2 mg/kg; LGC, mixed silage supplementation with lactic acid bacteria 5 mg/kg, glucose 30 g/kg and cellulase 2 mg/kg.

a: rapidly degradable fraction; b: slowly degradable fraction; a + b: total degradable fraction; c: degradation rate of slowly degradable fraction; ED: effective degradability.

In the same row, values with different letter mean significant differences (*P* < 0.05).

### 3.6 Ruminal crude protein degradation of mixed silage

The ruminal CP degradation of LGC group at 4 h and 24 h was higher (*P* < 0.05) than that of CON, LAB and GLU groups (**Table 5**). At 72 h, the CP degradation rate of all groups ranged from 74.52% to 80.40%. Similar to DM degradability, the CP degradation occurred mainly before 24 h. No significant difference (*P* > 0.05) of slowly degradable fraction was observed among all groups (**Table 6**). However, the rapidly degradable fraction of CEL and LGC groups was higher (*P* < 0.05) than that of other groups. Similarly, the total degradable fraction of CEL and LGC groups was significantly enhanced (*P* < 0.05) as compared to CON and GLU groups. Furthermore, the effective degradability of inoculated treatments was more than 60% and the effective degradability of LGC group was higher (*P* < 0.05) than CON, LAB and GLU groups.

**Table 5:** Rumen degradability of crude protein of mixed silage at different time points (%).

Time point (h)	Treatments					SEM	*P*-value
	CON	LAB	GLU	CEL	LGC		
4	35.89^b^	37.02^b^	34.99^b^	41.75^a^	40.55^a^	0.701	<0.001
8	48.68^ab^	48.34^ab^	45.31^b^	51.77^a^	54.32^a^	1.043	0.043
16	56.97^b^	59.17^b^	61.33^ab^	59.15^b^	64.98^a^	0.898	0.035
24	62.78^b^	65.61^b^	64.38^b^	68.57^ab^	71.04^a^	0.903	0.010
36	67.12^c^	68.71^bc^	69.22^bc^	71.79^ab^	73.32^a^	0.724	0.027
48	69.75	72.98	71.15	74.74	74.05	1.121	0.653
72	75.36^b^	77.54^ab^	74.52^b^	78.11^ab^	80.40^a^	0.688	0.033

CON, control group without additive; LAB, mixed silage inoculated with lactic acid bacteria 5 mg/kg; GLU, mixed silage supplementation with glucose 30 g/kg; CEL, mixed silage supplementation with cellulase 2 mg/kg; LGC, mixed silage supplementation with lactic acid bacteria 5 mg/kg, glucose 30 g/kg and cellulase 2 mg/kg.

In the same row, values with different letter mean significant differences (*P* < 0.05).

**Table 6:** Rumen degradation parameters of crude protein of mixed silage.

Items	Treatments					SEM	*P*-value
	CON	LAB	GLU	CEL	LGC		
a (%)	34.20^b^	36.40^b^	36.91^b^	40.19^a^	39.76^a^	0.623	0.001
b (%)	38.25	42.67	39.79	41.26	42.70	0.762	0.292
c (%/h)	0.051^c^	0.059^ab^	0.053^bc^	0.056^abc^	0.060^a^	0.001	0.024
a + b (%)	72.45^c^	79.06^ab^	76.69^b^	81.45^a^	82.46^a^	0.991	0.001
ED (%)	57.89^d^	64.20^bc^	61.92^c^	66.76^ab^	67.92^a^	0.913	<0.001

CON, control group without additive; LAB, mixed silage inoculated with lactic acid bacteria 5 mg/kg; GLU, mixed silage supplementation with glucose 30 g/kg; CEL, mixed silage supplementation with cellulase 2 mg/kg; LGC, mixed silage supplementation with lactic acid bacteria 5 mg/kg, glucose 30 g/kg and cellulase 2 mg/kg.

a: rapidly degradable fraction; b: slowly degradable fraction; a + b: total degradable fraction; c: degradation rate of slowly degradable fraction; ED: effective degradability.

In the same row, values with different letter mean significant differences (*P* < 0.05).

### 3.7 Ruminal neutral detergent fiber degradation of mixed silage

At 24 h, the NDF degradability of LAB, CEL and LGC groups was higher (*P* < 0.05) than that of other groups (**Table 7**). The ruminal NDF degradability of mixed silage in LGC group at 72 h was significantly increased (*P* < 0.05) when compared to CON, LAB and GLU groups. Unlike DM and CP degradability, the NDF degradation of mixed silage occurred mainly after 24 h. The rapidly degradable fraction of all groups was lower and it had no significant difference (*P* > 0.05) (**Table 8**). However, the slowly degradable fraction of LGC group was significantly increased (*P* < 0.05) as compared with CON, LAB and GLU groups. The NDF effective degradability of all treatments ranged from 29.07% to 34.50%. Moreover, the effective degradability of CEL and LGC groups was higher (*P* < 0.05) than other groups.

**Table 7:** Rumen degradability of neutral detergent fiber of mixed silage at different time points (%).

Time point (h)	Treatments					SEM	*P*-value
	CON	LAB	GLU	CEL	LGC		
4	7.17^c^	9.04^abc^	8.41^bc^	10.44^ab^	11.06^a^	0.431	0.012
8	13.73	14.30	13.49	17.02	15.65	0.469	0.070
16	18.14	20.37	17.36	19.69	21.09	0.514	0.103
24	27.05^b^	33.76^a^	27.68^b^	32.35^a^	33.50^a^	0.862	0.006
36	38.31^a^	40.51^a^	33.91^b^	39.71^a^	38.44^a^	0.719	0.017
48	45.34^bc^	47.83^abc^	44.80^c^	48.71^ab^	50.71^a^	0.683	0.016
72	47.52^c^	50.38^bc^	48.79^c^	52.96^ab^	55.11^a^	0.785	0.002

CON, control group without additive; LAB, mixed silage inoculated with lactic acid bacteria 5 mg/kg; GLU, mixed silage supplementation with glucose 30 g/kg; CEL, mixed silage supplementation with cellulase 2 mg/kg; LGC, mixed silage supplementation with lactic acid bacteria 5 mg/kg, glucose 30 g/kg and cellulase 2 mg/kg.

In the same row, values with different letter mean significant differences (*P* < 0.05).

**Table 8:** Rumen degradation parameters of neutral detergent fiber of mixed silage.

Items	Treatments					SEM	*P*-value
	CON	LAB	GLU	CEL	LGC		
a (%)	4.15	3.92	4.05	4.50	5.03	0.329	0.863
b (%)	44.52^c^	45.69^bc^	45.40^bc^	48.77^ab^	50.40^a^	0.708	0.018
c (%/h)	0.040	0.041	0.044	0.045	0.044	0.001	0.237
a + b (%)	48.67^b^	49.61^b^	49.44^b^	53.27^a^	55.43^a^	0.707	<0.001
ED (%)	29.07^b^	29.99^b^	30.60^b^	33.19^a^	34.50^a^	0.561	0.001

CON, control group without additive; LAB, mixed silage inoculated with lactic acid bacteria 5 mg/kg; GLU, mixed silage supplementation with glucose 30 g/kg; CEL, mixed silage supplementation with cellulase 2 mg/kg; LGC, mixed silage supplementation with lactic acid bacteria 5 mg/kg, glucose 30 g/kg and cellulase 2 mg/kg.

a: rapidly degradable fraction; b: slowly degradable fraction; a + b: total degradable fraction; c: degradation rate of slowly degradable fraction; ED: effective degradability.

In the same row, values with different letter mean significant differences (*P* < 0.05).

### 3.8 Ruminal acid detergent fiber degradation of mixed silage

The ADF degradability did not show significant difference (*P* > 0.05) among all groups from 4 h to 16 h (**Table 9**). At 24 h, the ADF degradability of GLU group was lower (*P* < 0.05) than other groups. In addition, compared with other groups, the ADF degradability at 72 h of CEL and LGC groups was significantly increased (*P* < 0.05). In accordance with NDF, the ADF of all mixed silage degraded slowly before 16 h; then from 16 h to 48 h, the degradation speed of all groups was faster. The rapidly degradable fraction of mixed silage was lower and the peak value was only 4.03% (**Table 10**). No significant difference (*P* > 0.05) of rapidly and slowly degradable fractions was found among all groups. The effective degradability of LGC group was maximum and higher (*P* < 0.05) than that of LAB and GLU groups.

**Table 9:** Rumen degradability of acid detergent fiber of mixed silage at different time points (%).

Time point (h)	Treatments					SEM	*P*-value
	CON	LAB	GLU	CEL	LGC		
4	8.14	6.01	5.16	6.54	8.05	0.467	0.180
8	13.03	9.12	9.59	11.11	11.07	0.478	0.060
16	15.04	13.22	12.04	13.70	13.98	0.437	0.295
24	22.86^a^	25.77^a^	18.54^b^	24.30^a^	22.55^a^	0.721	0.007
36	32.70	32.67	30.60	35.65	34.88	0.704	0.155
48	41.64^b^	40.15^b^	39.84^b^	46.60^a^	46.35^a^	0.902	0.009
72	43.14^b^	42.85^b^	42.65^b^	48.63^a^	50.28^a^	0.976	0.007

CON, control group without additive; LAB, mixed silage inoculated with lactic acid bacteria 5 mg/kg; GLU, mixed silage supplementation with glucose 30 g/kg; CEL, mixed silage supplementation with cellulase 2 mg/kg; LGC, mixed silage supplementation with lactic acid bacteria 5 mg/kg, glucose 30 g/kg and cellulase 2 mg/kg.

In the same row, values with different letter mean significant differences (*P* < 0.05).

**Table 10:** Rumen degradation parameters of acid detergent fiber of mixed silage.

Items	Treatments					SEM	*P*-value
	CON	LAB	GLU	CEL	LGC		
a (%)	4.03	3.55	2.93	3.06	3.62	0.246	0.663
b (%)	54.12	47.02	45.90	51.71	51.45	1.087	0.066
c (%/h)	0.022^c^	0.025^bc^	0.024^c^	0.031^ab^	0.034^a^	0.001	0.004
a + b (%)	58.15^a^	50.57^bc^	48.83^c^	54.76^ab^	55.07^ab^	1.069	0.021
ED (%)	26.51^abc^	24.73^bc^	22.62^c^	28.66^ab^	30.18^a^	0.809	0.007

CON, control group without additive; LAB, mixed silage inoculated with lactic acid bacteria 5 mg/kg; GLU, mixed silage supplementation with glucose 30 g/kg; CEL, mixed silage supplementation with cellulase 2 mg/kg; LGC, mixed silage supplementation with lactic acid bacteria 5 mg/kg, glucose 30 g/kg and cellulase 2 mg/kg.

a: rapidly degradable fraction; b: slowly degradable fraction; a + b: total degradable fraction; c: degradation rate of slowly degradable fraction; ED: effective degradability.

In the same row, values with different letter mean significant differences (*P* < 0.05).

## 4 Discussion

The forage grass characteristics directly affect the quality of silage. Our previous study found that the moisture content of amaranth was high, whereas the WSC content was low (Liu et al., 2017; Ma et al., 2019). Thus, the quality of amaranth silage alone was relatively low. The corn straw has high DM content. In the current study, we used corn straw as mixed material to improve the quality of amaranth silage by adding different additives. The chemical compositions affect feeding value of silage. After 60 days of ensiling, mixed silage inoculation with lactic acid bacteria significantly increased DM and CP contents. In the fermentation process of silage, a series of biochemical changes occur because of the action of a variety of microorganisms, resulting in nutrients loss (Zhang et al., 2022). A previous study found that the DM content of silage inoculated with *L. plantarum* was increased (Ren et al., 2020), which was consistent with our result. The reason may be related to less production of silage liquid by lactic acid bacteria action, which contains about 8% DM (Yang et al., 2020). However, the underlying mechanism of action still needs further study. Additionally, the inoculation with *L. plantarum* can promote homo-fermentation and prevent organic degradation caused by insufficient lactic acid yield (Ren et al., 2020), which are beneficial for reducing the loss of nutrients contents. Overall, the DM content of mixed silage of amaranth and corn straw was elevated as compared to individual amaranth silage (Liu et al., 2017). Corn straw is an effective mixed material to improve the quality of amaranth silage.

Research has reported that sufficient WSC concentration (≥ 5%) was necessary to ensure the silage quality (Zi et al., 2022a). The WSC was low in mixed materials; thus, we added glucose as additive to improve the silage quality. With the progress of silage fermentation, a large amount of WSC was consumed by lactic acid bacteria, leading to WSC content to below 3% after fermentation of 60 days. The cellulase can promote fermentation of lactic acid bacteria by degrading structural carbohydrates to provide substrate (Agustinho et al., 2021). Our results showed that mixed silage treated with cellulase (CEL and LGC groups) significantly decreased NDF and ADF contents when compared to CON and GLU groups. Similar result was found in Xiong et al. (2022) study, who reported that hybrid *Pennisetum* silage treated with cellulase reduced NDF and ADF contents. Three additives combinations showed highest DM and CP contents and lowest NDF content. This may be associated with synergistic action among different additives. The glucose and cellulase provided enough fermentation substrate for lactic acid bacteria to produce lactic acid, which were helpful for decreasing nutrients loss. In terms of chemical composition, the mixed silage of amaranth and corn straw inoculated with lactic acid bacteria, glucose and cellulase can improve nutritional value.

After 60 days of ensiling, pH in all groups reduced below 4.2, an important index that suggested silage was well preserved (McDonald et al., 1991). Compared with CON and LAB groups, the pH of LGC group was significantly reduced. The lactic acid bacteria can decrease the silage pH by utilizing WSC to produce lactic acid. Although the silage in LAB group was inoculated with lactic acid bacteria, the fermentation substrate was inadequate. Thus, the silage could not produce enough lactic acid to reduce pH, which was matched to the lactic acid result. The addition of glucose and cellulase provided substance for bacteria in the silage to promote fermentation process and was conducive to decreasing pH. A previous study in native grass silage found that the addition of lactic acid bacteria and molasses reduced pH and increased lactic acid content (Li et al., 2022), which was in accordance with our study.

High content of lactic acid content can inhibit the growth of harmful microorganisms, thus reducing the production of butyric acid (McDonald et al., 1991). In ruminants production, feeding silage with high butyric acid will increase the probability of metabolic disease such as ketosis in animals (Vicente et al., 2014). Butyric acid was detected in CON and CEL groups, which suggested that the mixed silage was contaminated by mold as seen by the microbial population results, and silage quality decreased. Propionic acid and butyric acid can consume some of the metabolic energy during production. The conversion of lactic acid to butyric acid leads to the loss of more than half of DM content (Zhao et al., 2022), which has negative effect on feed intake of animals. In the present study, mixed silage inoculated with additives significantly reduced propionic acid content. Besides, the acetic acid of CON group was higher than GLU, CEL and LGC groups. The lower WSC content in CON and LAB silage may accelerate the conversion from homo-fermentation to hetero-fermentation, contributing to higher acetic acid concentration in mixed silage of CON and LAB groups after ensiling was finished (Muck, 2013).

In general, NH_3_-N production is associated with CP breakdown caused by enzymes and microorganisms in silage and NH_3_-N/TN can be used to reveal the extent of proteolysis (Wang et al., 2019). Compared with CON group, inoculation with lactic acid bacteria, glucose and cellulase alone or in combination markedly reduced NH_3_-N/TN in mixed silage. Among them, the LGC group showed lowest NH_3_-N/TN, indicating that undesirable proteolytic bacteria were inhibited effectively in additives-treated silages. After 60 days of fermentation, the decline of NH_3_-N/TN in LGC group might be that the synergistic effect of different additives could cause nitrification, which transferred NH_3_-N to nitrate nitrogen (Fang et al., 2022). The future researches should be paid more attention to the effects of different additives on nitrogen transformation in fermentation process of amaranth silage. In the fermentation of silage, protein hydrolysis can be inhibited by acid environment, which prevents proteolytic enzyme activity and decreases NH_3_-N concentration (Li et al., 2018c). In our study, combined addition of lactic acid bacteria, glucose and cellulase could maintain lowest acid environment (pH=3.78) to maximum the NH_3_-N reduction of mixed silage, which was line with CP result. The results mentioned earlier suggested that different additives in the fermentation process had the ability to improve fermentation quality, and the combined inoculation efficiently promoted the fermentation.

The production of NH_3_-N and organic acids is closely related to microbial population. In the current experiment, the lactic acid bacteria count was significantly increased in LAB and LGC groups. Correspondingly, the harmful microorganisms counts, including aerobic bacteria and mold, were reduced. Moreover, inoculation of glucose also had significant reduction of aerobic bacteria and mold counts. The additional supplementation of lactic acid bacteria and an enough supply of carbohydrate source as substrates could explain the decrease in harmful microorganisms counts (So et al., 2020). Previously, a study has found that the combination of lactic acid bacteria and molasses can inhibit growth of yeast, mold and coliform bacteria in soybean silage (Ni et al., 2017), which was basically consistent with our study. The positive effects were most likely because of the additive’ ability to produce sufficient lactic acid to reduce pH and create acid experiment, thus preventing the growth of a variety of unwanted microorganisms in silage fermentation process (Henderson, 1993). In silage, yeast, mold and coliform bacteria play a critical role in butyric acid production by secreting amino acid decarboxylases to generate butyric acid (Jia et al., 2021). Therefore, higher these microorganisms counts in CON and CEL groups induced increased butyric acid, which was not beneficial for silage quality. Overall, these results verified that additives can reduce the number of undesirable microorganisms in mixed silage of amaranth and corn straw, with the LGC groups showing the greatest influence.

After exposure to air, the aerobic microorganisms activity of silage begin to enhance and release a mass of heat because of metabolizing and consuming nutrients, resulting in increased pH and nutrients loss (Yuan et al., 2015). Thus, the change of temperature is usually used as an important parameter to evaluate the silage aerobic stability. In our study, combination of all additive significantly lengthened the aerobic stability. Particularly, yeast is deemed to be the promoter of aerobic spoilage, the number of which is closely related to high temperature of silage (He et al., 2020). The improvement in aerobic stability of LGC group could be attributed to a reduction of yeast count. After 5 days of aerobic exposure, the number of lactic acid bacteria was increased and the aerobic bacteria and mold counts were decreased in additives treatments. The possible reason was mainly because although the lactic acid bacteria activity of additives group was not insufficient to maintain aerobic stability long time after mixed silage was opened, it could still prevent the growth of harmful microorganisms via maintaining the acid environment and antibacterial substances generation within a short period (da Silva et al., 2018). In ruminants’ production, coliform bacteria can cause diseases such as diarrhea and mastitis, which lead to considerable economic damage (Ursula et al., 2022). After aerobic exposure, coliform bacteria of all group was increased as compared with ensiling stage, but the CON group had highest counts. In short, inoculation with silage can improve the aerobic stability of mixed silage of amaranth and corn straw.

In addition to fermentation quality and chemical composition, the utilization rate of nutrients by animals is a critical index to assess the silage quality. Therefore, we used dairy cows as experimental animals to investigate the effects of different additives on the ruminal degradation characteristics of mixed silage composed of amaranth and corn straw. In dairy cows’ production, dry matter intake is important to maintain lactation and the ruminal DM degradability is positively related to dry matter intake (Hao et al., 2020). In our study, the LGC group had the highest DM degradation at 72 h and effective degradability. A previous study reported that the addition of lactic acid preparation can improve the DM digestibility (Rowghani and Zamiri, 2009), which was in accordance with our result. This result may be related to higher DM content in LGC group. Similarly, the ruminal CP degradation of LGC group showed greatest effects. The CP degradation rate is affected by true protein concentration and amino acid composition proportion of CP in the feedstuff (Cardozo et al., 2004). The combined inoculation of additives may have positive effects on the amino acid composition of mixed silage, but it needs future study. In addition, the CP total degradable fraction of LGC and CEL groups exceeded than that of CON and GLU groups. A plausible explanation for this result was that the mixed silage inoculated with cellulase contained high content of soluble true protein including the form of non-ammonia N (Wang et al., 2022a), as seen by the NH_3_-N result mentioned earlier.

Increased crude fiber degradation rate is conducive to rumen fermentation and can result in elevated contents of volatile fatty acids that provide more energy for dairy cows to maintain production performance (Abbasi et al., 2018). Previous researchers have found that the addition of additive enhanced the ruminal NDF and ADF degradation of silage (Jiao et al., 2021; Wang et al., 2022b), which were consistent with our results. Furthermore, inoculation of cellulase showed significant improvement of ruminal degradability of NDF and ADF. The result was likely attributed to the breakdown of connection between polyester and cellulose by cellulase treatment, which could enable the degradation and utilization of structural carbohydrates by microbiota in the rumen (Li et al., 2018a). In the future, metagenomics and other multi-omics technologies can be used to explore effects of different additives on the microbial community and function in mixed silage of amaranth and corn straw.

## 5 Conclusions

The results obtained from current study provided evidence that the lactic acid bacteria inoculation increased DM, CP and lactic acid contents and decreased propionic acid content and aerobic bacteria and mold counts of mixed silage. Glucose addition had no significant effect on the chemical composition, where increased lactic acid content and decreased aerobic bacteria and mold counts of mixed silage. Cellulase treatment reduced NDF and ADF contents as well as NH_3_-N/TN and mold count. Combining the three additives contributed in a number ways of mixed silage quality, including promotion in the fermentation, reduction in the harmful bacteria counts and improvement of aerobic stability and nutrients composition. In addition, the rumen degradation of nutrients was improved in inoculation of three additives.

## Data availability statement

The original contributions presented in the study are included in the article/**Supplementary Material**. Further inquiries can be directed to the corresponding author/s.

## Ethics statement

The animal study was reviewed and approved by the Institutional Animal Care and Use Committee of Guangdong Ocean University (Zhanjiang, Guangdong, China).

## Author contributions

JM, XF and SG conceived and designed the research. JM, XF, ZM, XH, and MT performed the experiment and sample analysis. JM, XF, and ZZ analyzed the data. JM and XF wrote the original manuscript. JM, XF, FY, and SG reviewed the manuscript. All authors contributed to the article and approved the submitted version.
