# Supplementary material for: Silage additives improve fermentation quality, aerobic stability and rumen degradation in mixed silage composed of amaranth and corn straw
Source: Front Plant Sci. 2023 Jun 22;14:1189747. doi: 10.3389/fpls.2023.1189747 (PMC10325724; doi:10.3389/fpls.2023.1189747)
Supplement: Supplementary file 1 [file Table_1.docx]

Supplementary Material

[Silage additives](javascript:;) improve fermentation quality, aerobic stability and rumen degradation in mixed silage composed of amaranth and corn straw

Jian Ma^1†^, Xue Fan^1,2†^, Zhuang Ma^2^, Xiuwen Huang^2^, Minghuan Tang^2^, Fuquan Yin^1^, Zhihui Zhao^1^, Shangquan Gan^1*^

*** Correspondence:** Shangquan Gan: gansq1977@126.com

# Supplementary Tables

Table S1 Chemical composition of amaranth and corn [straw](javascript:;) (DM basis, %).

| Items | DM | CP | OM | WSC | NDF | ADF |
| --- | --- | --- | --- | --- | --- | --- |
| Amaranth | 19.15 | 10.38 | 87.16 | 4.17 | 56.58 | 38.57 |
| Corn straw | 90.43 | 7.18 | 90.55 | 3.71 | 70.59 | 41.69 |

DM, dry matter; CP, crude protein; OM, [organic](E:/%E6%9C%89%E9%81%93/Dict/8.5.1.0/resultui/html/index.html#/javascript:;) [matter](E:/%E6%9C%89%E9%81%93/Dict/8.5.1.0/resultui/html/index.html#/javascript:;); WSC, [water soluble carbohydrate](javascript:;); NDF, neutral detergent fiber; ADF, acid detergent fiber.

Table S2 Feed ingredients and nutrient composition of experimental diet (DM basis).

| Ingredients, % |  | Nutrient levels, % |  |
| --- | --- | --- | --- |
| Alfalfa hay | 10.28 | NE_L_^2)^, MJ/kg | 5.11 |
| Chinese wildrye | 14.40 | CP | 12.86 |
| Whole corn silage | 21.92 | NDF | 45.37 |
| Amaranth silage | 13.40 | ADF | 30.26 |
| Steam-flaked corn | 15.32 | EE | 2.51 |
| Soybean meal | 6.48 | Ca | 0.67 |
| [Cottonseed](E:/%E6%9C%89%E9%81%93/Dict/8.9.4.0/resultui/html/index.html#/javascript:;) [meal](E:/%E6%9C%89%E9%81%93/Dict/8.9.4.0/resultui/html/index.html#/javascript:;) | 4.44 | P | 0.38 |
| Wheat bran | 6.75 |  |  |
| DDGS | 4.66 |  |  |
| Limestone | 0.80 |  |  |
| NaCl | 0.38 |  |  |
| NaHCO_3_ | 0.75 |  |  |
| Premix^1)^ | 0.42 |  |  |

DM, dry matter; DDGS, distillers dried grains with soluble; NE_L_, net energy for lactation; CP, crude protein; NDF, neutral detergent fiber; ADF, acid detergent fiber; EE, Ether extract.

^1)^ The premix provided the following per kilogram of the diet: VA 800 000 IU, VD 1 200 IU, VE 50 IU, Fe, 100 mg, Zn 60 mg, Mn 40 mg, Cu 10 mg, I 0.50 mg, Se 0.30 mg, Co 0.10 mg.

^2)^ NE_L_ was a calculated value, while others were measured values.
